# Supplementary material for: Traveling Waves Enhance Transport during Cytoplasmic Streaming in Drosophila Oogenesis
Source: ArXiv. 2026 Sep 22:arXiv:2609.26656v1. Preprint. [Version 1] (PMC13622435)
Supplement: Supplementary file 1 [file NIHPP2609.26656v1-supplement-1.pdf]

Supporting Information

Traveling Waves Enhance Transport during Cytoplasmic Streaming in *Drosophila* Oogenesis

Olenka Jain, David B. Stein, Reza Farhadifar, Elizabeth R. Gavis, Stanislav Y. Shvartsman, Michael J. Shelley

Correspondence: Michael J. Shelley
mshelley@flatironinstitute.org

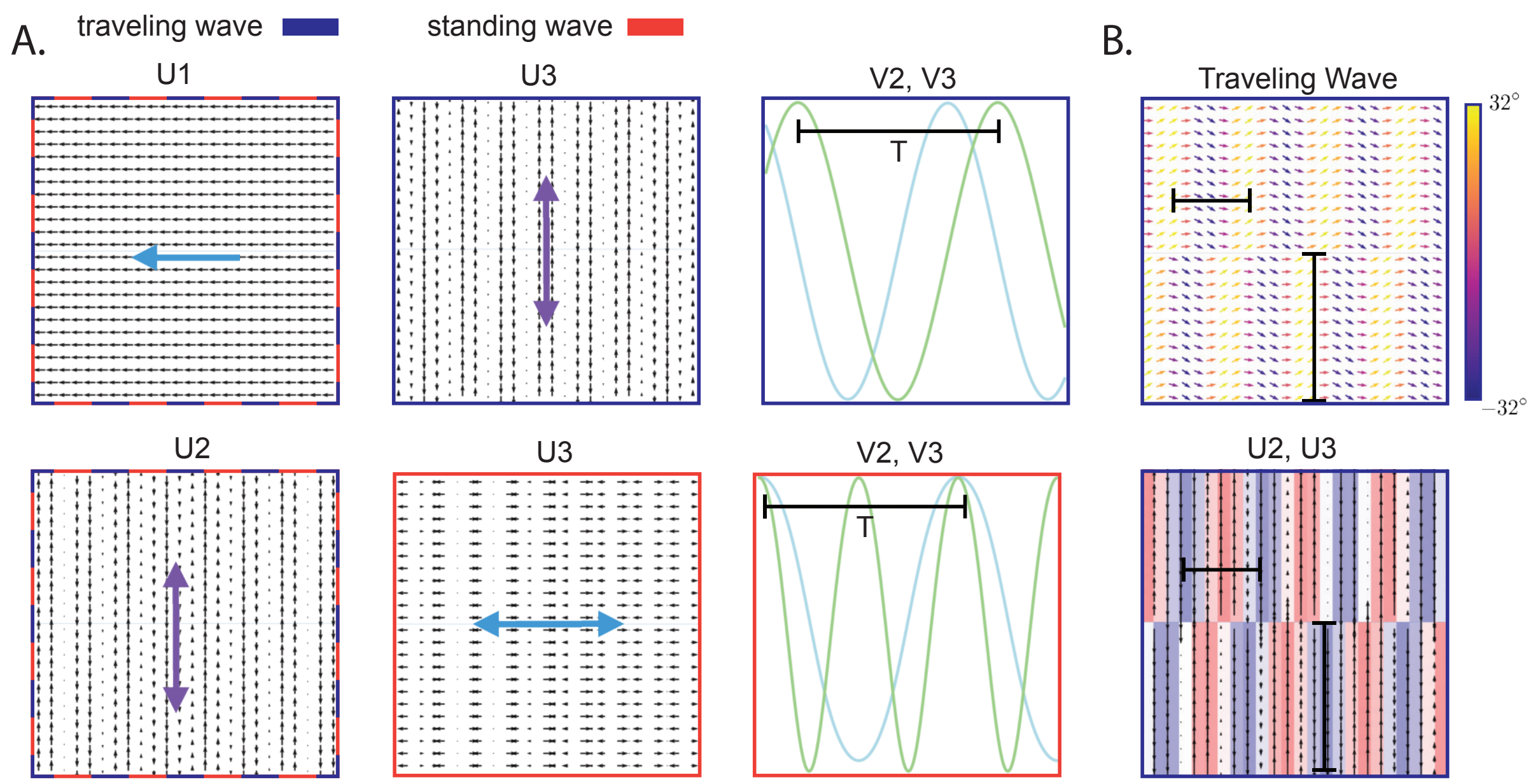

Figure 6: A. Singular value decomposition performed on representative traveling and standing waves. For both a low amplitude/high wavelength traveling wave and a low amplitude/high wavelength standing wave, the first spatial mode, U_1, is a vector in the direction of travel, $\vec{\mathbf{v}}$. For both wave types, U_2 alternates between patches of $\vec{\mathbf{v}}+\frac{\pi}{2}$ and $\vec{\mathbf{v}}-\frac{\pi}{2}$. The difference between wave types appears in U_3. For the traveling wave, U_3 is a phase shift of U_2; for the standing wave, U_3 has vectors parallel or anti-parallel to $\vec{\mathbf{v}}$. The time dependent amplitudes likewise reveal the difference between traveling and standing waves. For the traveling wave, V_2 and V_3 have equal period and are $\pi/2$ out of phase. For a standing wave, V_3 is double the period of V_2. For both types of waves, the period is given by V_2; for a traveling wave this can be derived analytically using a small angle approximation. B. For a traveling wave, the size of the $\vec{v}+\frac{\pi}{2}$ and $\vec{v}-\frac{\pi}{2}$ regions gives the wavelength of the underlying traveling wave, and the length of that patch is set by the phase.

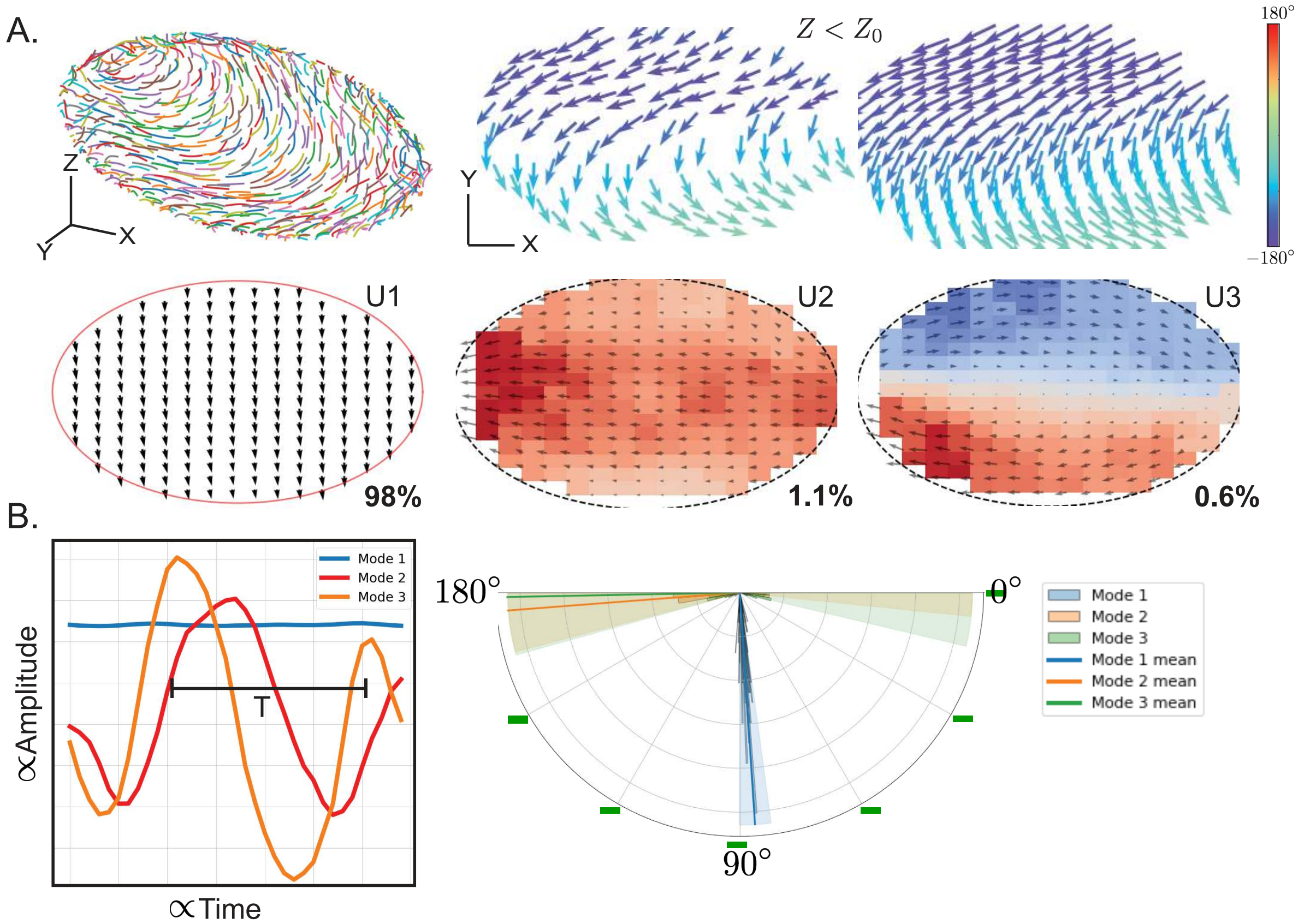

Figure 7: A. Singular Value Decomposition performed on a simulated microscopy slice. Fibers from the 3D simulation below some Z height were projected onto the $X-Y$ plane where the $X-$axis was the long-axis of the ellipsoid. The fiber angle was interpolated onto a structured grid and used as input for the SVD. The results showed consistency with the experiments both in the structure of the spatial modes. The first mode explained over 90% of the variance and contained vectors uniformly in the direction of solid body rotation (up to a sign change). The next two spatial modes were about equal in their importance, with vectors perpendicular to the first mode and oscillating time dependent amplitudes which were $\frac{\pi}{2}$ out of phase, but had the same period. The analysis of spatial structure in the top three modes is summarized by the circular histogram.

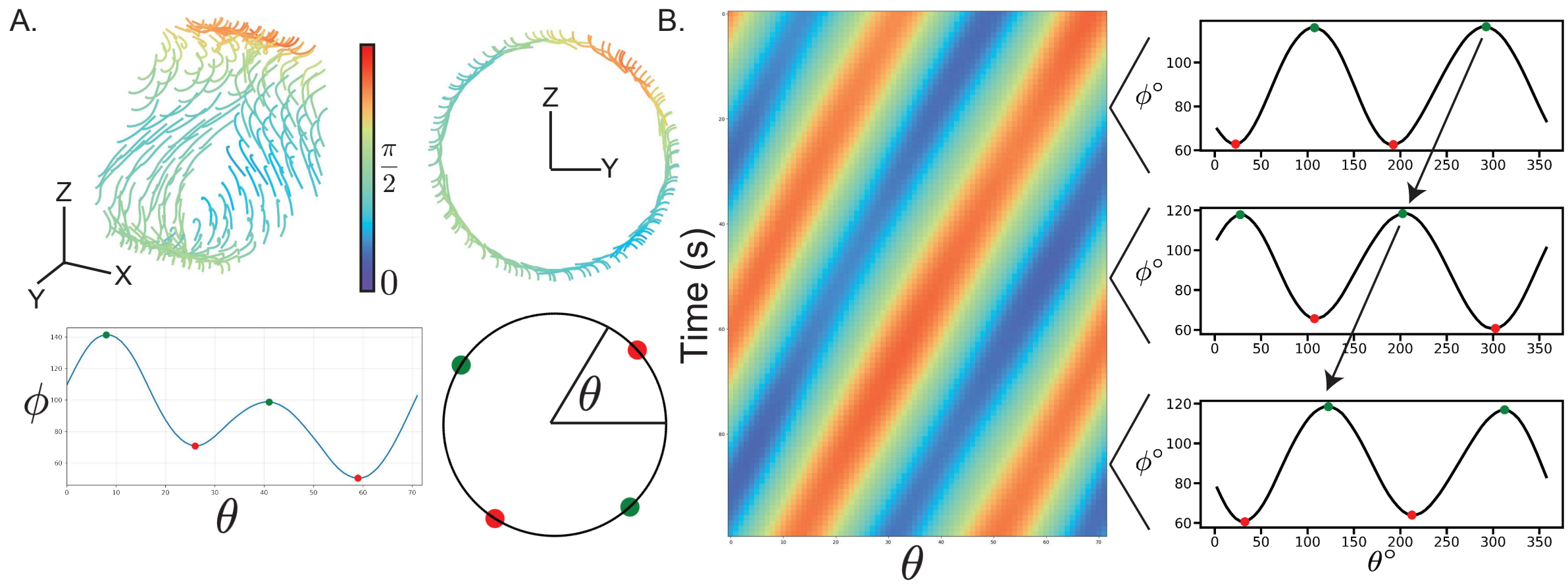

Figure 8: A. The center belt of fibers from $-1 < X < 1$ was used to characterize the traveling wave in all ellipsoids, whose long axis was set as the $X-$axis. The average angle of each fiber relative to the long axis, ϕ, was plotted as a function of θ of the cross-sectional circle. The peaks and troughs are shown in red and green. B. A kymograph of was generated where the maximum and minimum of the color bar are set by the maximum and minimum angle deviation from $\frac{\pi}{2}$.

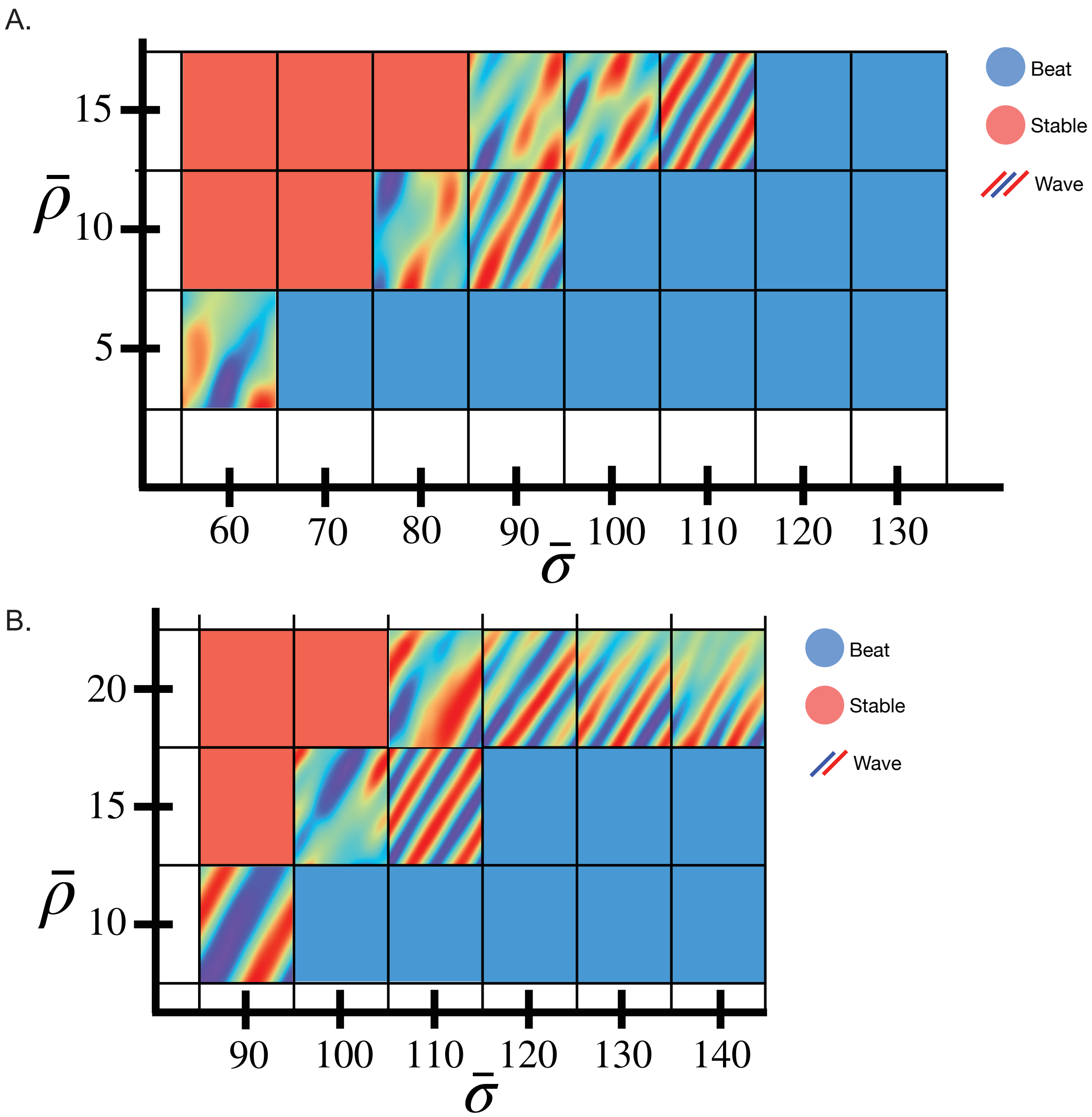

Figure 9: A. Simulations were run across a range of $\bar{\rho}$ and $\bar{\sigma}$ parameters for an ellipsoid with semi-major and semi-minor axes: $A = 5, B = 3, C = 3$. Waves are illustrated by kymographs. B. Simulations were run across a range of $\bar{\rho}$ and $\bar{\sigma}$ parameters for an ellipsoid with semi-major and semi-minor axes: $A = 7, B = 3, C = 3$. For both ellipsoids, waving dynamics were found between the stable and beating regimes.

Supporting Movies

Movie S1. Waving dynamics in a Stage 10B oocyte (1) visualized with Jupiter-GFP line.

Movie S2. Waving dynamics in a Stage 10B oocyte (2) visualized with Jupiter-GFP line.

Movie S3. Waving dynamics in a Stage 12 oocyte visualized with Jupiter-GFP line.

Movie S4. Waving dynamics in a Stage 10B oocyte about to dump where the defect has moved from the poles and the waves appear more consistent with waves on the edge of streaming and beating.

Movie S5. Radial transport in simulations with $\bar{\rho} = 15$ and $\bar{\sigma} = 50$, in the steady streaming regime, with a bi-toroidal component pushing particles near the pole inward along the axis. Tracer particles are initialized randomly using rejection sampling, with $-1 \leq x \leq 1$, $r > 2.5$, and within the ellipsoid (i.e. near the center along the axis and close to the periphery). Their motion is visualized over one hour.

Movie S6. Radial transport in simulations with $\bar{\rho} = 15$ and $\bar{\sigma} = 80$, in the steady streaming regime, with a bi-toroidal component pushing particles near the pole outward along the periphery. Tracer particles are initialized randomly using rejection sampling, with $-1 \leq x \leq 1$, $r > 2.5$, and within the ellipsoid (i.e. near the center along the axis and close to the periphery). Their motion is visualized over one hour.

Movie S7. Radial transport in simulations with $\bar{\rho} = 15$ and $\bar{\sigma} = 120$, in the waving regime. Tracer particles are initialized randomly using rejection sampling, with $-1 \leq x \leq 1$, $r > 2.5$, and within the ellipsoid (i.e. near the center along the axis and close to the periphery). Their motion is visualized over one hour.

Movie S8. Radial transport in simulations with $\bar{\rho} = 15$ and $\bar{\sigma} = 200$, in the beating regime. Tracer particles are initialized randomly using rejection sampling, with $-1 \leq x \leq 1$, $r > 2.5$, and within the ellipsoid (i.e. near the center along the axis and close to the periphery). Their motion is visualized over one hour.

Movie S9. Axial transport in simulations with $\bar{\rho} = 15$ and $\bar{\sigma} = 50$, in the steady streaming regime, with a bi-toroidal component pushing particles near the pole inward along the axis. Tracer particles are initialized randomly using rejection sampling, with $x > -4$ and within the ellipsoid (i.e. near the left cap). Their motion is visualized over one hour.

Movie S10. Axial transport in simulations with $\bar{\rho} = 15$ and $\bar{\sigma} = 80$, in the steady streaming regime, with a bi-toroidal component pushing particles near the pole outward along the periphery. Tracer particles are initialized randomly using rejection sampling, with $x > -4$ and within the ellipsoid (i.e. near the left cap). Their motion is visualized over one hour.

Movie S11. Axial transport in simulations with $\bar{\rho} = 15$ and $\bar{\sigma} = 120$, in the waving regime. Tracer particles are initialized randomly using rejection sampling, with $x > -4$ and within the ellipsoid (i.e. near the left cap). Their motion is visualized over one hour.

Movie S12. Axial transport in simulations with $\bar{\rho} = 15$ and $\bar{\sigma} = 120$, in the beating regime. Tracer particles are initialized randomly using rejection sampling, with $x > -4$ and within the ellipsoid (i.e. near the left cap). Their motion is visualized over one hour.
